# Purpura Fulminans following Thermal Injury

**DOI:** 10.1155/2013/782386

**Published:** 2013-05-09

**Authors:** Jiongyu Hu, Xupin Jiang, Ting He, Qizhi Luo

**Affiliations:** Institute of Burn Research, Southwest Hospital, The Third Military Medical University, Shapingba District, Chongqing 400038, China

## Abstract

Purpura fulminans is a rare syndrome of intravascular thrombosis and hemorrhagic infarction of the skin, which is an unusual cutaneous manifestation of disseminated intravascular coagulation. It often occurs in small children and babies due to infection and/or sepsis, rarely in adults in clinic. We report the first case of deadly purpura fulminans following thermal injury in a 64-year-old Chinese woman. The purpura developed sharply and aggravated multiple organ dysfunction. The patient died of purpura fulminans, disseminated intravascular coagulation, and multiple organ dysfunction syndrome.

## 1. Introduction

Purpura fulminans (PF) is a life-threatening hemorrhagic condition characterized by acute onset hypotension, fever, cutaneous hemorrhage, and necrosis [1]. It is often caused by the infection of *Staphylococcus aureus*, hemolytic streptococcus and meningococcus [2], and can be classified into three distinct categories: inherited or acquired abnormalities of protein C or other coagulation systems, acute infectious PF, and idiopathic PF [3]. PF often occurs in small children and babies due to infection and/or sepsis, rarely in adults following thermal injury. In this report, we describe a case of PF following alcohol flame thermal injury.

## 2. Case Presentation

**Figure 1:**
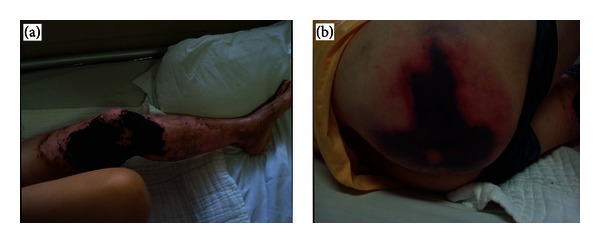
Photograph of the patient when admitted to the department. (a) Alcohol flame burn covered by traditional Chinese medicine. (b) Painful erythema on the right buttocks.

A 64-year-old Chinese woman was admitted to our department with an 18-day history of alcohol flame burn and a 12-hour painful erythema on the right buttocks. Physical examination showed a low-grade fever (37.8°C), burn wounds (2% TBSA) on the left inner thigh covered by traditional Chinese medicine (Figure 1(a)), and petechial rash on the right buttocks (Figure 1(b)). Past medical history and family history were unremarkable. Laboratory findings include haemoglobin anomaly (95 g/L), leukocytosis (15.38 × 10^9^/L), thrombocytopenia (90 × 10^9^/L), and prolonged APTT (69.5 s). Tests of kidney and liver function and abdominal ultrasound showed no abnormality of note.

The purpura developed sharply and occurred on the lower extremities in the next morning. Laboratory tests showed decreased platelet (21 × 10^9^/L), haemoglobin (57 g/L), fibrinogen (0.82 g/L, normal more than 1.8 g/L) and factor VIII activity (29.1%, normal 70%–150%), increased leukocytosis (18.09 × 10^9^/L), D-dimer (30018 ng/mL, normal less than 392 ng/mL), and APTT (67.8 s). Thrombophilia screens displayed low antithrombin III activity (57.1%, normal 75%–125%) but normal protein C and S concentrations. There were no abnormalities in prothrombin time, thrombin time, C3, C4, anticardiolipin, antinuclear, and IgG or IgA antibody levels.

**Figure 2:**
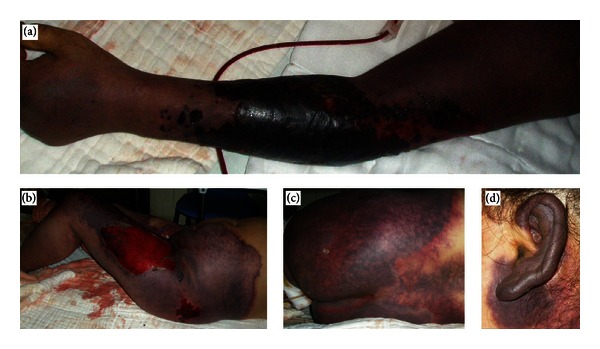
Photograph of the sharply developed purpura. Purpuric rash in the (a) left arm, (b) left lower limbs, (c) buttocks and (d) left ear.

The patient was diagnosed with PF and received antibiotics (imipenem, cilastatin sodium, and ticarcillin), fluid resuscitation, and component blood transfusion (fresh frozen plasma and blood platelets) but complicated by new emergence and enlargement of rash, which gradually enlarged into an irregular pattern of full-thickness skin and soft-tissue loss (Figures 2(a)–2(d)). She received bilateral lower extremities escharotomy but developed acute renal failure with anuria 24 h later. Test of kidney and liver function revealed increased creatinine (201 umol/L), glutamic-pyruvic transaminase (391.8 IU/L), and glutamic oxaloacetic transaminase (939.8 IU/L). The blood routine test revealed decreased platelet (11 × 10^9^/L), haemoglobin (40 g/L), and red blood cell (1.34 × 10^12^/L). 

The patient received methylprednisolone treatment, but it did not work. The purpuric rash covered approximately 70% TBSA in the next afternoon, and the platelet count was 9 × 10^9^/L, haemoglobin was 31 g/L, and leukocytosis was 27.28 × 10^9^/L. Test of kidney and liver function revealed increased creatinine (308 umol/L), glutamic-pyruvic transaminase (1354 IU/L), and glutamic-oxalacetic transaminase (4009 IU/L). Myocardial enzyme levels, including creatine kinase and lactic dehydrogenase, were increased notably. The bacterial cultivation indicated *Staphylococcus epidermidis* and *Enterobacter cloacae* in burn wound but not in blood, which were sensitive to multiple antibiotics including imipenem, cilastatin sodium, and ticarcillin. Consequently, the patient died on the sixth day after admission of purpura fulminans, disseminated intravascular coagulation, and multiple organ dysfunction syndrome.

## 3. Discussion

PF is a lethal haemorrhagic condition usually associated with infection and/or sepsis. It occurs mainly in babies and small children, and the mortality rate in the acute phase ranges between 18% and 40% [4]. It is most commonly associated with *Neisseria meningitidis* sepsis, and proteins C and S and antithrombin III are always involved in the coagulopathy pathogenesis [5, 6]. DIC is believed to be the major pathophysiological mechanism [7]. The abnormal APTT, platelet count, and fibrinogen point towards ongoing coagulopathy as negative prognostic markers.

The mainstays of primary management are to remove the underlying cause of clotting abnormalities and with supportive treatment. Protein C supplementation could decrease mortality and morbidity [8], but the safety and efficacy of heparin therapy has not been confirmed [9]. The attention is turned to skin loss management after acute phase, including debridement and grafting, such as escharotomies, fasciotomies, or major amputations. Early escharotomy is recommended in case of amputation [10]. In this case, the PF started by the time that the burn injury approximately healed, and the *Staphylococcus epidermidis* and *Enterobacter cloacae*, which were the pathogens of PF, were detected on the wound but not in the blood. Although we used the sensitive antibiotics the first time, the remarkably impressive coagulopathy was ultimately uncontrollable. We suspected that there appears to be a preference for heparin application in cases of ongoing coagulopathy, especially in adults in which the possibility of hereditary proteins S and C deficiency is little.
